# Diagnosis of Secondary Sclerosing Cholangitis by Port Site Metastasis

**DOI:** 10.7759/cureus.18177

**Published:** 2021-09-21

**Authors:** David S Braun, Bryce Bushe, Prashant Kedia, Paul Tarnasky

**Affiliations:** 1 Internal Medicine, Methodist Dallas Medical Center, Dallas, USA; 2 Gastroenterology, Methodist Health System, Dallas, USA

**Keywords:** obstructive jaundice, ercp, extrahepatic biliary malignancy, port site metastasis, secondary sclerosing cholangitis

## Abstract

Port site metastasis is an uncommon but challenging pathological entity whereby metastatic cancer is discovered at the operative port site after surgery. Secondary sclerosing cholangitis is a multifocal stricture disease of the biliary system as the result of extra-biliary pathology; rarely, it is due to an infiltrative disorder such as neoplasia. This is the first reported case of secondary sclerosing cholangitis that was diagnosed with metastatic cancer following the discovery of port site metastasis after laparoscopic cholecystectomy.

## Introduction

Port site metastasis (PSM) is a well-documented postoperative phenomenon in which cancer is discovered at port incision sites after laparoscopic surgery. Secondary sclerosing cholangitis (SSC) is characterized by multifocal biliary strictures as the result of a known nonbiliary pathology. We present herein a unique case of a young patient that presented with multifocal biliary strictures following laparoscopic cholecystectomy (LCCX). The diagnosis of SSC due to metastatic malignancy was ultimately made only after discovering PSM.

## Case presentation

A 28-year-old Hispanic female was referred back to our institution from an outside facility because of right upper quadrant abdominal pain, jaundice, and fever. She had undergone a robotic-assisted LCCX 11 months prior for symptomatic cholelithiasis. Preoperative bile duct imaging and serum liver chemistries were normal. Gallbladder pathology reported gallstones, chronic cholecystitis, and no evidence of neoplasia.

**Figure 1:**
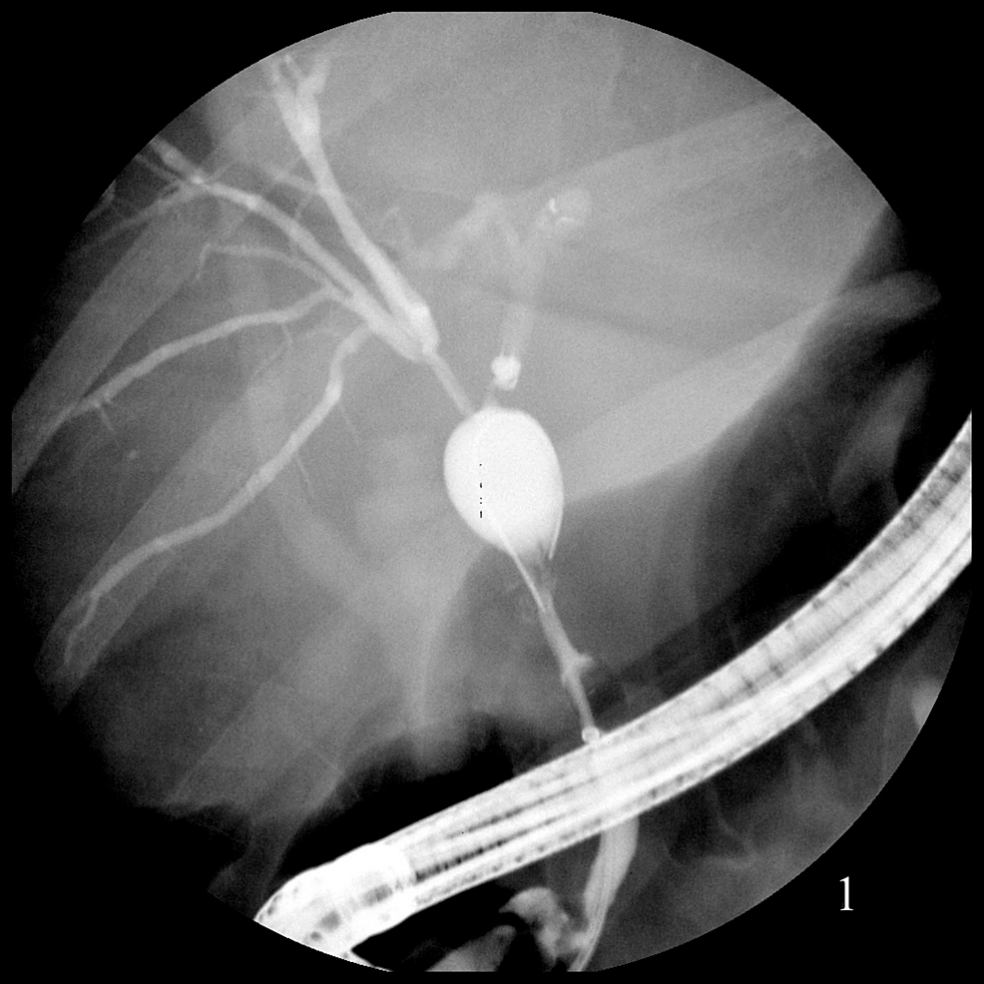
Cholangiogram demonstrating multifocal intra and extrahepatic biliary strictures.

She was first referred to our institution about six months after LCCX with abdominal pain, jaundice, and pruritus. Initial endoscopic retrograde cholangiopancreatography (ERCP) revealed multifocal extrahepatic and intrahepatic hilar strictures with focal saccular dilation of the common hepatic duct (Figure 1). Cholangioscopy was required to achieve guidewire access of the left and right hepatic ducts via very tight focal strictures and bilateral 8.5F stents were placed. The strictures appeared extrinsic without evidence of mass lesions; cholangioscopy-directed intraductal biopsies revealed necrosis with atypical epithelial cells. Serologic workup including IgG-4 was within normal limits. Hepatobiliary surgery was consulted. An abdominal CT-angiogram was negative for visceral vascular pathology (i.e. postoperative vascular injury).

Given her age and the multi-focal nature of the disease, the patient was started on an empiric trial with prednisone for possible autoimmune sclerosing cholangitis. Repeat ERCP one month later demonstrated near resolution of strictures and no residual waist on fully inflated balloons, so a stent-free trial was planned. However, the patient returned within one week with worsening abdominal pain and recurrent jaundice that required repeat ERCP with stent placements.

**Figure 2:**
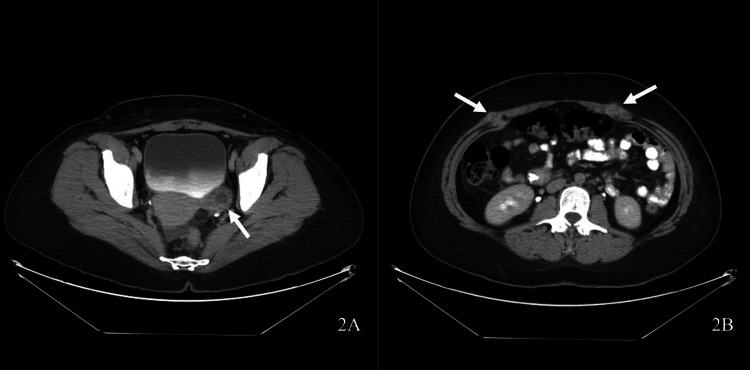
Axial abdominal CT scan demonstrating (A) a complex left adnexal mass and (B) trocar implants consistent with port site metastasis (arrows).

**Figure 3:**
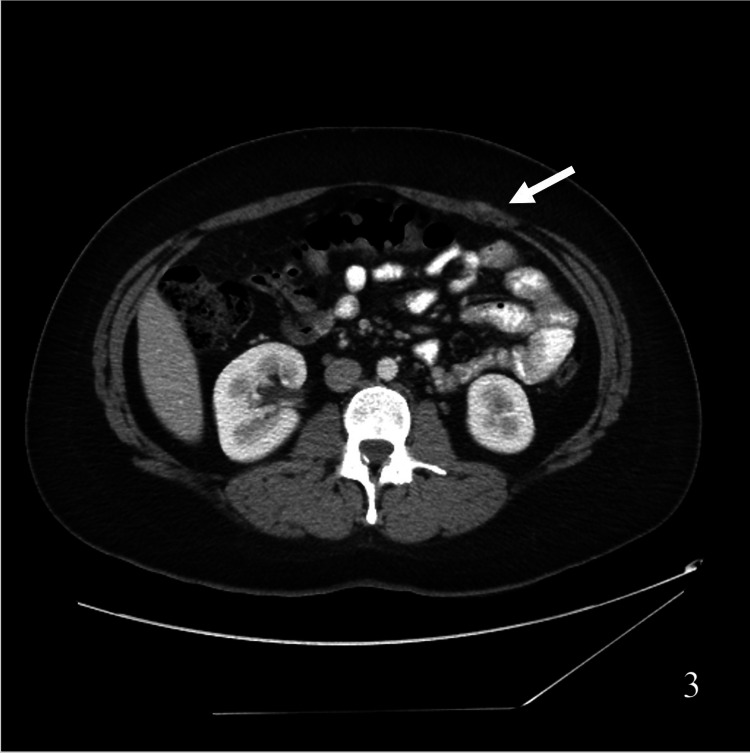
Axial abdominal CT scan demonstrating evidence of very early (three months) port site metastases after cholecystectomy.

**Figure 4:**
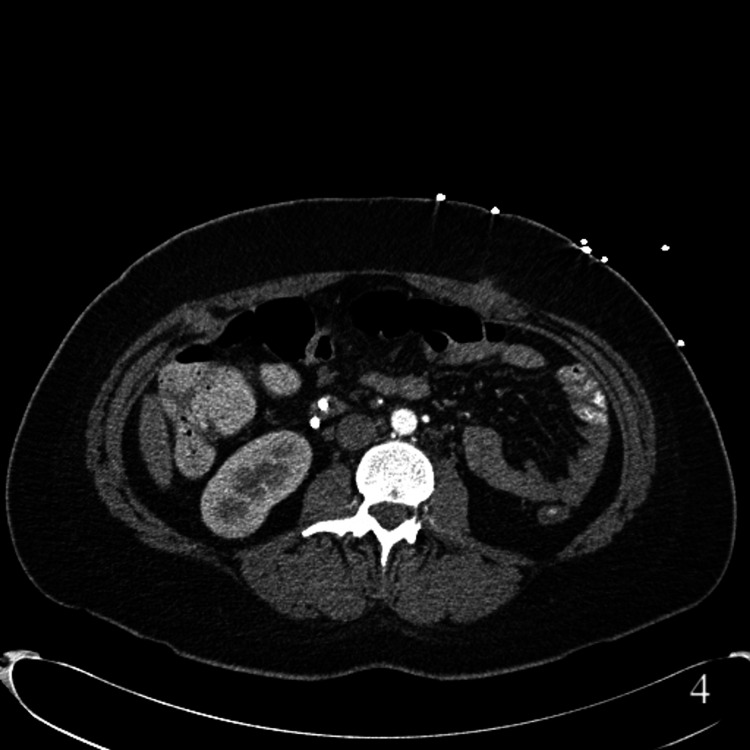
Axial abdominal CT scan demonstrating evidence of port site metastases eight months after cholecystectomy.

She continued to experience recurrent symptoms and presented again 11 months post-LCCX. An abdominal CT scan revealed a new mass in the porta hepatis, omental caking, bilateral adnexal enlargement, and a large pelvic mass (Figure 2 a). In addition, masses were now noted bilaterally on the anterior abdominal wall at the laparoscopic port sites suspicious for PSM (Figure 2 b). Upon repeat review of previous abdominal imaging, subtle nodularity of the port sites on prior CT scans was noted. Axial images from three (Figure 3) and eight (Figure 4) months post-LCCX all showed findings consistent with PSM. Serum cancer antigen 19-9 and cancer antigen 125 levels were elevated (8080 U/mL and 202 U/mL, respectively). Diagnostic laparoscopy demonstrated ascites and peritoneal carcinomatosis. Liver wedge, omental biopsies, and ascites cytology revealed adenocarcinoma. Histologic staining could not differentiate between a hepatobiliary or mucinous ovarian neoplasm as the primary tumor site. She was referred to oncology and underwent chemotherapy. Unfortunately, the patient died 15 months post-LCCX. 

## Discussion

Most cases of SSC are due to autoimmune, toxic, ischemic, or infectious etiologies; rarely, infiltrative disorders such as cancer cause SSC [1]. Biliary strictures related to SSC mimic primary sclerosing cholangitis clinically but the clinical outcomes are worse [2]. Numerous underlying etiologies of SSC have been described, with the most common being immune-mediated IgG4-related disease.

Diffuse multifocal biliary strictures from SSC due to malignancy is a very uncommon phenomenon. Case reports have documented SSC secondary to lymphoma as well as metastatic prostate, ovarian, and gallbladder cancers [3-6]. Lew et al. documented complete resolution of SSC findings via ERCP after successful treatment of metastatic ovarian cancer [7].

The majority of PSM occurs in patients with cancer either known at the time of surgery, discovered during surgery, or identified postoperatively following pathologic review [8]. Most commonly, PSM after laparoscopy (1-4%) is in patients with metastasis from known colorectal or gynecologic primaries [9]. Among patients with known gynecologic cancers, the overall incidence of PSM is about 2%; PSM risk is higher (6%) in patients with ovarian cancer [10]. There are also reports of PSM after laparoscopic diagnosis of ovarian tumors thought to be of low malignant potential [11]. Gynecologic cancers, more specifically ovarian cancers, have been associated with short intervals from surgery to PSM [8]. However, there is also a report of PSM from ovarian cancer two years after a normal diagnostic laparoscopy was performed for pelvic pain [12].

Following LCCX, PSM is most often related to the delayed spread of gallbladder cancer that is only discovered postoperatively and less commonly due to colon, pancreatic, or ovarian cancer [13]. One case of PSM after LCCX due to cholangiocarcinoma was reported where the patient presented with a single focus of distal bile duct obstruction [14].

Occult ovarian cancer-associated PSM presentation post-LCCX can be obscure. Carlson et al. reported a case of PSM 22 months after LCCX for symptomatic gallstones; there was no evidence of cancer at the time of the LCCX [15]. Ovarian cancer was diagnosed six months later and treated with a complete response, but the patient presented with PSM 16 months after her ovarian cancer diagnosis. Another report described PSM four months after LCCX [16]. A microscopic focus of cancer was detected in the gallbladder specimen so it was initially believed to be unsuspected gallbladder cancer. However, ovarian cancer was diagnosed during the investigation of the PSM.

While the source of primary cancer was not clearly defined in our case, hepatobiliary or ovarian sources were considered most likely. We are aware of only a few other reported cases of PSM in which the primary malignancy was never discovered [17-19]. For example, a 50-year-old female presented with PSM 18 months after an elective LCCX for symptomatic gallstones [17]. Tumor immunohistochemistry studies were consistent with an ovarian primary, but no evidence of ovarian tumor was found during an exploratory laparotomy. Polychronidis et al. reported a 75-year-old male with PSM (well-differentiated extra-hepatic mucinous adenocarcinoma) 11 months after LCCX without a known primary malignancy; magnetic resonance cholangiopancreatography (MRCP) and ERCP were used to search for extrahepatic malignancy, but results were normal [18]. A mucin-secreting papillary adenocarcinoma PSM was diagnosed in a 45-year-old female 28 months after LCCX [19]. Pathology of the gallbladder specimen did not reveal any malignancy and the patient’s operative report did not mention any omental nodularity, unexplained ascites, or obvious primary masses to suggest metastatic disease at the time of her surgery.

In this case, there was a subtle progression in the nodular appearance of our patient’s port sites on cross-sectional imaging over time that was not noted until the patient developed large hepatic and ovarian masses. Unfortunately, PSM portends a poor prognosis so earlier PSM detection would not likely have provided any chance for surgical cure. The two-year survival rate for patients diagnosed with PSM secondary to gallbladder cancer is <20% [13]. It is likely that PSM secondary to gynecologic malignancies is even more aggressive as they often present with shorter intervals (e.g., eight days) between laparoscopy and PSM [20].

## Conclusions

Unique from the previously reported cases of PSM, our patient presented with multifocal biliary strictures and symptomatic jaundice. In this case, operative bile-duct injury after a complicated LCCX and autoimmune SSC was considered the most likely diagnosis. Retrospective review of imaging for PSM in cases of unexplained bile duct strictures may prove helpful for early diagnosis of SSC. Unfortunately, PSM portends a poor prognosis so earlier PSM detection likely would not have provided any chance for surgical cure. In summary, PSM due to an unknown primary is exceedingly rare in itself. This is the first reported case, to our knowledge, of PSM with an unknown primary that presented with SSC after an LCCX.
